# Effect of Hurricane Katrina on Arboviral Disease Transmission

**DOI:** 10.3201/eid1308.061570

**Published:** 2007-08

**Authors:** Jennifer A. Lehman, Alison F. Hinckley, Krista L. Kniss, Roger S. Nasci, Theresa L. Smith, Grant L. Campbell, Edward B. Hayes

**Affiliations:** * Centers for Disease Control and Prevention, Fort Collins, Colorado, USA

**Keywords:** Hurricane Katrina, West Nile virus, Saint Louis Encephalitus virus, arboviral, neuroinvasive, letter

**To the Editor:** Rarely has the aftermath of a natural disaster in the continental United States resulted in increased transmission of mosquitoborne viruses (*1*). However, on August 29, 2005, Hurricane Katrina struck Louisiana and Mississippi, where mosquito-borne West Nile (WNV) and St. Louis encephalitis viruses are endemic.

Using data from the ArboNET system of the Centers for Disease Control and Prevention, we evaluated the short-term effects of Hurricane Katrina on the reported incidence of human West Nile neuroinvasive disease (WNND) and Saint Louis encephalitis (SLE) in Louisiana and Mississippi using the reported week of onset and the year (2003–2005). We also evaluated incidence by onset date and county (or parish) over 3 time intervals (January 1–August 31, September 1–September 30, and October 1–October 30) in 2005. Reporting lag was evaluated by onset dates and corresponding dates of reports. Because the completeness of reporting of West Nile fever and other arboviral fever cases is highly variable, only reports of human WNND and SLE were considered.

In Louisiana, the highest reported incidence of WNND occurred in the second week of August 2005, before Hurricane Katrina made landfall. Although the number of cases reported in 2005 (117) was higher than in 2003 (85) or 2004 (101), the number of cases peaked during roughly the same weeks in each year. In Mississippi, the total number of cases reported in 2005 (39) was only slightly higher than in previous years (31 in 2004 and 34 in 2003). The number of cases peaked in mid-September 2005, later than the peak in 2004, but similar to when a second peak occurred in 2003. Thus, the increase in WNND incidence for either state does not appear to be hurricane-related.

In Louisiana, 82 WNND cases in 20 parishes had onset between January 1, 2005 and August 31, 2005. In comparison, 25 WNND cases had onset between September 1, 2005 and September 30, 2005; a total of 14 of these cases in 7 parishes had not reported WNND cases previously in 2005; a total of 5 of these parishes had detected WNV activity in animals before the hurricane. From October 1, 2005 to October 31, 2005, a total of 10 additional WNND cases were reported in Louisiana, including 1 case from a parish that had not previously reported cases. Only 5 cases with illness onset after the hurricane resided in coastal parishes. In Mississippi, 17 WNND cases in 10 counties had onset between January 1, 2005 and August 31, 2005. Twenty cases had onset between September 1, 2005 and September 30, 2005, including 9 cases in 4 counties that had not reported WNND cases previously in 2005; a total of 2 of these counties had detected WNV activity in animals before the hurricane. From October 1, 2005 to October 31, 2005 2 more WNND cases were reported in Mississippi, including 1 from a county that had not reported cases previously. All cases with illness onset after the hurricane resided in inland counties. Thus, in both states the coastal counties and parishes that were hardest hit by the hurricane had the fewest number of posthurricane WNND cases.

In 2005, Louisiana reported 2 SLE cases and Mississippi reported 5. Both Louisiana cases and 4 of the Mississippi cases had onset of illness after September 1. In 2004, no SLE cases had been reported by either of these states. In 2003, a total of 9 cases were reported in Louisiana (3 with onset in September) and 2 in Mississippi (with onsets in May and June). Thus, Hurricane Katrina did not appear to increase SLE incidence in Louisiana, and if it did increase incidence in Mississippi, the increase was minimal.

In both 2003 and 2004, Louisiana’s median reporting time to ArboNET was ≈30 days. In 2005, the median reporting time prehurricane was 36 days and posthurricane was 69 days. Louisiana state officials believed that this reporting lag was largely due to impaired transport and collection of biologic samples and relocation of diagnostic facilities immediately following the hurricane. In contrast, in 2003 and 2004, Mississippi’s median reporting time to ArboNET was 21 days and 36 days, respectively. In 2005, the median reporting time prehurricane was 23 days and posthurricane was 14 days. Mississippi state officials believed that the improved reporting time was due to the additional help and longer hours worked by health department officials following the hurricane.

Although Hurricane Katrina disrupted WNV surveillance in Louisiana, it did not appear to increase the incidence of WNND and SLE in either Louisiana or Mississippi. In coastal areas, the hurricane destroyed housing and impeded vector control, thus possibly increasing the risk of mosquito-borne infections (*1**,**2*). However, hurricane-force winds and heavy flooding might have actually decreased the risk of WNV and SLE transmission by dispersing or killing birds and mosquitoes, and destroying their habitat. Many people were promptly evacuated to less affected areas, where, on the basis of previous years’ data showing seasonality of WNV transmission, the risk of infection was probably decreasing. Natural disasters do not usually cause an immediate increase in arboviral diseases (*1**,**2*). However, if hurricanes strike early in transmission season, there could be a late increase in risk after vector and host populations are re-established. In addition, risk could increase when people are relocated to areas where transmission is intense.
